# Rural, Urban and Migrant Differences in Non-Communicable Disease Risk-Factors in Middle Income Countries: A Cross-Sectional Study of WHO-SAGE Data

**DOI:** 10.1371/journal.pone.0122747

**Published:** 2015-04-07

**Authors:** Oyinlola Oyebode, Utz J. Pape, Anthony A. Laverty, John T. Lee, Nandita Bhan, Christopher Millett

**Affiliations:** 1 Department of Primary Care and Public Health, Imperial College, Reynolds Building, St Dunstand’s Road, London, United Kingdom; 2 Public Health Foundation of India, New Delhi, India; Institute of Infectious Disease and Molecular Medicine, SOUTH AFRICA

## Abstract

**Background:**

Understanding how urbanisation and rural-urban migration influence risk-factors for non-communicable disease (NCD) is crucial for developing effective preventative strategies globally. This study compares NCD risk-factor prevalence in urban, rural and migrant populations in China, Ghana, India, Mexico, Russia and South Africa.

**Methods:**

Study participants were 39,436 adults within the WHO Study on global AGEing and adult health (SAGE), surveyed 2007–2010. Risk ratios (RR) for each risk-factor were calculated using logistic regression in country-specific and all country pooled analyses, adjusted for age, sex and survey design. Fully adjusted models included income quintile, marital status and education.

**Results:**

Regular alcohol consumption was lower in migrant and urban groups than in rural groups (pooled RR and 95%CI: 0.47 (0.31–0.68); 0.58, (0.46–0.72), respectively). Occupational physical activity was lower (0.86 (0.72–0.98); 0.76 (0.65–0.85)) while active travel and recreational physical activity were higher (pooled RRs for urban groups; 1.05 (1.00–1.09), 2.36 (1.95–2.83), respectively; for migrant groups: 1.07 (1.0 -1.12), 1.71 (1.11–2.53), respectively). Overweight, raised waist circumference and diagnosed diabetes were higher in urban groups (1.19 (1.04–1.35), 1.24 (1.07–1.42), 1.69 (1.15–2.47), respectively). Exceptions to these trends exist: obesity indicators were higher in rural Russia; active travel was lower in urban groups in Ghana and India; and in South Africa, urban groups had the highest alcohol consumption.

**Conclusion:**

Migrants and urban dwellers had similar NCD risk-factor profiles. These were not consistently worse than those seen in rural dwellers. The variable impact of urbanisation on NCD risk must be considered in the design and evaluation of strategies to reduce the growing burden of NCDs globally.

## Introduction

Non-communicable diseases (NCDs) are the leading cause of global disease burden,[1, 2] with 80% of NCD mortality occurring in low- and middle-income countries (LMICs).[3] In some regions, particularly Sub-Saharan Africa, as the burden of NCD grows, resources for treatment may not meet population needs. This is recognised as a priority issue with a political declaration signed by all the UN Member States in 2011, committing them to the prevention and control of NCDs.[4] On this basis, the WHO developed a global target to reduce mortality from cardiovascular disease, cancer, diabetes and chronic respiratory diseases by 25% before 2025 (known as the 25x25 target).[5]

The rising prevalence of NCDs is taking place in the context of a rapidly urbanising population. The world’s urban population has been growing at an average of 2.6% a year and is expected to increase to 6.3 billion people, 70% of the total population by 2050.[6] The majority of this growth will be concentrated in LMICs: Asia is projected an urban population increase of 1.4 billion, Africa 0.9 billion, and Latin America and the Caribbean 0.2 billion.[6] One of the key drivers of this trend is internal rural-urban migration, predominantly undertaken for economic reasons. For example, in China 200 million rural-urban migrants are anticipated between 2010 and 2020.[7]

Rural and urban living can determine health through a range of environmental, social and cultural factors. Both an ‘urban penalty’ suggested by nineteenth century research on infectious diseases (likely mediated through higher population density), and an ‘urban advantage’, suggested by 20^th^ century research into infant and child mortality and nutritional status (likely mediated by superior access to health services) have been proposed.[8] In addition, the “healthy migrant hypothesis” suggests that although migrants may face some disadvantages in their new living environment, they are likely to be a ‘selected’ population with good health pre-migration.[9] With respect to NCDs, there is concern that urbanisation and rural-urban migration may increase population exposure to risk-factors. This may be due to several factors including differential exposure to motorised travel and pollution, occupational physical activity, marketing and access to tobacco, alcohol and processed food products.[10–15] A recent systematic review of existing studies on NCD risk in rural, migrant and urban populations, found a gradient for most of the commonly reported NCD risk-factors with higher risk in migrants in comparison with rural dwellers, and lower risk in migrants in comparison with urban dwellers.[16] However, studies included in the review had important limitations, including small sample sizes, highly selected populations (e.g.: migrants to a single city, from a single ethnic group or within a particular occupational group, e.g.: factory workers), an absence of data on behavioural risk-factors and lack of adjustment for other factors (for example age, income, education and marital status). In addition, the review omitted examination of lifestyle factors including smoking, despite this having one of the largest population-attributable risks for NCDs.[17, 18]

There continues to be a dearth of nationally representative data on NCD risk-factors in LMICs, and limited opportunities to conduct cross-national studies to inform global health policy. This is an important knowledge gap given the 25x25 target. The aim of this study is to compare the prevalence of NCD risk-factors in urban, rural and rural-urban migrant groups in six middle income countries using nationally representative, cross-national data.

## Methods

### Participants and data

Study participants were adults over 18 years old within the WHO Study on global AGEing and adult health (SAGE), surveyed between 2007 and 2010 (Wave 1) in China, Ghana, India, Mexico, Russia and South Africa.

SAGE uses a clustered household sampling strategy designed to generate nationally representative cohorts. Although the primary purpose of the SAGE survey was to explore the health of aging populations, and as such more data is collected for individuals over 50 years than for adults aged 18–49 years, weighting adjusts the analyses to give nationally representative estimates based on the age-sex population structure. One household questionnaire was completed for each selected household in face-to-face interviews and individual questionnaires were collected from one randomly selected individual aged 18–49 years and all individuals aged over 50 years, including by proxy where an individual was unable to complete the questionnaire. Individual response rates are as follows: 53% Mexico, 68% India, 75% South Africa, 81% in Ghana, 83% Russia and 93% in China. Further details of SAGE have been published elsewhere.[19] A physical examination was used to collect height, weight, waist circumference and blood pressure.

Participant’s place of residence was classified as urban or rural on the basis of country specific definitions [Table 1]. Participants were considered “urban” if they were a current resident of an urban area and had lived there all their life, or reported living only in other urban areas previously; participants were considered “rural” if they were a current resident of a rural area and had lived there all their life, or reported living only in other rural areas previously; participants were considered “migrant” if they were a current resident of an urban area and if they reported that either their previous place of residence was rural, or that they had lived most of their adulthood or childhood in a rural area. Urban to rural migrants were excluded from this study as there were too few to make an independent category for analysis and the population group is small and unlikely to grow, so they are not currently a significant concern to health policy-makers.

**Table 1:** 

Country	Definition of urban or rural
China	Defined by administrative district in the national sampling frame.
Ghana	Rural area is defined as consisting of <5000 inhabitants.
India	Rural areas defined by status as "villages", Urban areas defined by status as "wards".
Mexico	Rural area: < 2500 inhabitants; Urban area (city): >10,000 inhabitants; semi urban area: >2500–99 999 inhabitants.
Russia	Urban settlements are defined as legally established populated areas such as cities, towns and urban-type settlements (industrial communities, recreation zones, summer cottages). All remainder settlements are considered as rural ones. The category of cities (towns) in the Russian Federation includes, as a rule, settlements with at least 12 thousand inhabitants of whom not less than 85 per cent consist of workers, employees and their family members. The rules and criteria for definition of towns may be different in some regions.
South Africa	Defined by area type and service provision urban formal, urban informal, rural formal and rural informal. Semi-urban areas are classified as rural. An urban area is one which has been legally proclaimed as being urban e.g. towns, cities and metropolitan areas. See Statistics South Africa for more information.

### Ethics Committee Approval

The study is based on a secondary data set with no identifiable information on the survey participants. This dataset is available in the public domain for research use and hence no formal approval from the institutional review board is required. So, no ethics statement is required for this work.

The SAGE study received human subjects testing and ethics council approval from research review boards local to each participating site (China Center for disease control and prevention ethical review committee; University of Ghana Medical School Ethics and Protocol Review Committee; Institutional Review Board, International Institute for Population Sciences, India; Comisión de Ética en Investigación, Instituto Nacional de Salud Publica; Department of Prophylactic Medicine, Russian Academy of Medical Sciences; Human Sciences Research Council Ethics Committee, South Africa) and from the WHO Ethical Review Committee. Written informed consent was obtained from each respondent before interview and examination.

### Outcome Measures

Outcome measures were categorised as binary variables and included the following behavioural and clinical risk-factors: 1. ever and current tobacco smoking, 2. regular alcohol consumption (at least weekly) 3. recommended fruit and vegetable consumption (5+ portions/day), 4. recommended physical activity levels (75 minutes of vigorous or 150 minutes of moderate exercise/week) through either work, leisure or active travel 5. overweight (BMI ≥25 kg/m2), obesity (BMI ≥30 kg/m2), raised waist circumference (≥80cm for women, ≥94cm for men), 6. hypertension (systolic blood pressure ≥140 mmHg, diastolic ≥90 mmHg or on blood pressure medication) and 7. doctor-diagnosed diabetes. Outcome measures 1–4 and 7 were based on self-report. Outcome measures 5 and 6 were based on physical direct measurement.

### Statistical Analysis

Odds ratios and 95% confidence intervals (CI) were calculated for each risk-factor using logistic regression adjusted for survey design. We calculated age and sex adjusted odds ratios and fully adjusted odds ratios with the rural category as the reference group. Fully adjusted models included income quintile, categorised on the basis of the country specific distribution of income, marital status (currently married or not) and education (primary school or less, secondary school, higher education). Odds ratios were converted into risk ratios (RR), to aid interpretation, as described in Zhang 1998.[20] Analyses were run for individual countries and then data were pooled. Pooled analyses were conducted by normalising the country specific weights across the dataset and including country fixed-effects in the model. Statistical analysis was carried out using STATA 12.1.

## Results

### Participant Characteristics

39,436 participants were included in the study, with the largest number coming from China (14,261) followed by India (10,725), Ghana (4,579), Russia (3,973), South Africa (3,350) and Mexico (2,548). The mean age of participants was 52.9 years, ranging from 50.0 years in India to 62.9 years in Mexico. 43.8% of the total sample were men, ranging from 35.6% in Russia to 53.0% in Ghana [Table 2].

**Table 2:** Characteristics of the respondents and the frequency of health behaviours by country.

Country	China	Ghana	India	Mexico	Russia	South Africa	Total
**Sample Size**	14,261	4,579	10,725	2,548	3,973	3,350	39,436
**Age 18–39**	726 (5.1, *29*.*1*)	392 (8.6, *39*.*9*)	3,121 (29.1, *49*.*5*)	218 (8.6, *51*.*5*)	231 (5.8, *37*.*9*)	165 (4.9, *42*.*4*)	4853 (12.3, *39*.*0*)
**Age 40–59**	6,398 (44.9, *56*.*6*)	1,844 (40.3, *45*.*4*)	4,162 (38.8, *37*.*9*)	600 (23.6, *34*.*8*)	1,474 (37.1, *39*.*6*)	1,448 (43.2, *45*.*1*)	15,926 (40.4, *46*.*4*)
**Age 60+**	7,137 (50.0, *14*.*4*)	2,343 (51.2, *14*.*8*)	3,442 (32.1, *12*.*7*)	1,723 (67.6, *13*.*4*)	2,268 (57.1, *22*.*5*)	1,737 (51.9, *12*.*5*)	18,650 (47.3, *14*.*7*)
**Men (n, (%, *adj%*))**	6,903 (48.4, *52*.*1*)	2,425 (53.0, *50*.*9*)	4,239 (39.5, *51*.*9*)	975 (38.3, *47*.*7*)	1,415 (35.6, *45*.*5*)	1,296 (38.7, *45*.*1*)	17,253 (43.8, *50*.*9*)
**Urban (n (%, *adj%*)))**	5, 859 (41.1, *42*.*2*)	1,673 (36.5, *40*.*5*)	2,148 (20.0, *21*.*6*)	1,560 (61.2, *69*.*1*)	2,297 (57.8, *68*.*0*)	2,084 (62.2, *64*.*1*)	15,621 (39.6, *37*.*9*)
**Migrant (n (%, *adj%*)))**	1,210 (8.5, *6*.*9*)	373 (8.2, *9*.*5*)	639 (6.0, *4*.*6*)	368 (14.4, *9*.*8*)	837 (21.1, *15*.*8*)	241 (7.2, *12*.*3*)	3,668 (9.3, *7*.*3*)
**Rural (n (%, *adj%*)))**	7, 192 (50.4, *50*.*9*)	2,533 (55.3, *50*.*0*)	7,938 (74.0, *73*.*8*)	620 (24.3, *21*.*1*)	839 (21.1, *16*.*2*)	1,025 (30.6, *23*.*6*)	20,147 (51.1, *54*.*8*)
**Current smoking (n (%, *adj%*)))**	3, 891 (27.3, *32*.*4*)	551 (12.0, *8*.*4*)	4,158 (38.8, *42*.*7*)	479 (18.8, *25*.*3*)	748 (18.8, *28*.*7*)	866 (25.9, *25*.*9*)	10,693 (27.1, *35*.*5*)
**Ever smoking (n (%, *adj%*)))**	4,707 (33.0, *35*.*9*)	1,093 (23.9, *15*.*8*)	4,523 (42.2, *45*.*0*)	952 (37.4, *41*.*0*)	1,201 (30.2, *41*.*2*)	1,146 (34.2, *33*.*3*)	13,622 (34.5, *39*.*8*)
**Alcohol consumption > weekly (n (%, *adj%*)))** ^#^	2,111 (14.9, *16*.*3*)	1,012 (22.6, *22*.*1*)	393 (3.7, *4*.*9*)	83 (3.3, *2*.*5*)	263 (6.7, *8*.*9*)	268 (8.6, *10*.*0*)	4,130(10.6, *10*.*6*)
**Fruit + vegetable consumption < 5 portions (n (%, *adj%*)))**	1,531 (10.7, *7*.*1*)	3,224 (70.4, *68*.*3*)	9,590 (89.4, *89*.*3*)	2,032 (79.8, *67*.*9*)	3,215 (80.9, *70*.*9*)	2,394 (71.5, *64*.*2*)	21,986 (55.8, *51*.*1*)
**Occupational PA < 150 minutes (n (%, *adj%*)))**	7,798 (54.7, *45*.*8*)	1,280 (28.0, *24*.*0*)	2,996 (27.9, *25*.*0*)	1,576 (61.9, *61*.*1*)	1,151 (29.0, *19*.*9*)	2,171 (64.8, *51*.*4*	16,972 (43.0, *34*.*0*)
**Leisure PA <150 minutes (n (%, *adj%*)))**	12,139 (85.1, *83*.*9*)	3,952 (86.3, *83*.*4*)	9,377 (87.4, *81*.*3*)	2,392 (93.9, *90*.*8*)	3,605 (90.7, *76*.*1*)	3,138 (93.7, *86*.*9*)	34,603 (87.7, *82*.*0*)
**Active Travel <150 minutes (n (%, *adj%*)))**	3,134 (22.0, *25*.*7*)	807 (17.6, *18*.*8*)	2,732 (25.5, *22*.*0*)	598 (23.5, *23*.*5*)	637 (16.0, *13*.*1*)	593 (17.7, *15*.*1*)	8,501 (21.6, *22*.*2*)
**Overweight: BMI** **≥** **25 (n (%, *adj%*)))** ^**#**^	4,460 (32.7, *32*.*0*)	1,307 (29.2, *35*.*1*)	1,491 (14.1, *12*.*1*)	1,713 (72.8, *77*.*3*)	2,633 (67.8, *51*.*4*)	2,259 (69.2, *61*.*3*)	13,863 (36.3, *27*.*4*)
**Obesity: BMI** **≥** **30 (n (%, *adj%*)))** ^**#**^	744 (5.5, *4*.*9*)	465 (10.4, *13*.*2*)	383 (3.6, *3*.*2*)	763 (32.4, *28*.*1*)	1,177 (30.3, *18*.*9*)	1,350 (41.3, *30*.*5*)	4,882 (12.8, *7*.*0*)
**Raised waist circumference:** **≥** **94cm for men,** **≥** **80 for women (n (%, *adj%*)))**	5,948 (41.7, *37*.*6*)	1,779 (38.9, *39*.*8*)	3,478 (32.4, *24*.*8*)	2,086 (81.9, *69*.*8*)	2,971 (74.8, *51*.*2*)	2,155 (64.3, *52*.*6*)	18,417 (46.7, *34*.*7*)
**Hypertension: Systolic** **≥** **140, diastolic** **≥** **90 or currently on an antihypertensive (n (%, *adj%*)))**	8,310 (58.3, *41*.*3*)	2,508 (54.8, *44*.*2*)	2,971 (27.7, *21*.*1*)	1,581 (62.1, *33*.*8*)	2,585 (65.1, *39*.*6*)	2,559 (76.4, *53*.*0*)	20,514 (52.0, *33*.*4*)
**Doctor-diagnosed diabetes (n (%, *adj%*)))** ^**#**^	850 (6.0, *2*.*9*)	158 (3.5, *2*.*0*)	522 (4.9, *3*.*1*)	462 (18.1, *9*.*4*)	321 (8.2, *3*.*2*)	305 (9.4, *3*.*3*)	2,618 (6.7, *3*.*0*)

Adj% is the percentage when adjusting for survey design (using weights to give a nationally representative sample).

^#^ Denominators may not equal total sample size due to missing data for some variables.

51.1% of our study sample were rural dwellers, 39.6% were urban and 9.3% were rural-urban migrants. Within the individual countries, South Africa had the largest percentage of urban dwellers (62.2%), followed by Mexico (61.2%) and Russia (57.8%), while India had the highest percentage of rural participants (74.0%). Russia had the largest percentage of migrant participants (21.1%), followed by Mexico (14.4%) and China (8.5%).

The prevalence of NCD risk-factors varied considerably by country: E.g. the prevalence of current smoking ranged from 12.0% in Ghana to 38.8% in India; prevalence of raised waist circumference ranged from 32.4% in India to 81.9% in Mexico [Table 2].

### Comparisons between migrant, urban and rural groups

#### Smoking

In the age and sex adjusted model the six country pooled analyses showed lower current and ever smoking prevalence in urban and migrant groups compared with the rural group although this only achieved statistical significance in the urban group (RR 0.82 (0.70–0.93) for current smoking; RR 0.83 (0.73–0.94) for ever smoking). These differences attenuated in fully adjusted models such that current and ever smoking rates were similar in rural, urban and migrant groups.

Current smoking was lower in urban and migrant groups than in rural groups in China, Ghana and India. This contrasts with Mexico where there was significantly higher current and ever smoking rates in urban and migrant groups compared with the rural group. In South Africa the migrant group had a lower current and ever smoking rate (RR 0.41 (0.15–1.05) current smoking; RR 0.39 (0.15–0.89) ever smoking) but the urban group had similar rates to the rural group [S1 Fig; Table 3].

**Table 3:** Risk ratios for smoking variables in migrant and urban population groups with rural population groups as the reference category.

	Model 1: Adjusted for age and sex				Model 2: Adjusted for age, sex, income quintile, marital status and education			
	Current smoking		Ever smoking		Current smoking		Ever smoking	
	Migrant	Urban	Migrant	Urban	Migrant	Urban	Migrant	Urban
China	0.78 (0.54–1.07)	0.73 (0.56–0.94)**	0.88 (0.65–1.14)	0.72 (0.56–0.91)***	0.84 (0.59–1.14)	0.79 (0.62–1.00)**	0.90 (0.66–1.17)	0.73 (0.57–0.93)***
Ghana	0.35 (0.13–0.87)**	0.49 (0.30–0.80)***	0.68 (0.38–1.15)	0.74 (0.52–1.05)*	0.50 (0.19–1.28)	0.70 (0.44–1.09)	0.86 (0.49–1.40)	0.93 (0.65–1.32)
India	0.91 (0.72–1.11)	0.64 (0.53–0.78)***	0.89 (0.71–1.08)	0.65 (0.54–0.79)***	0.98 (0.81–1.16)	0.79 (0.66–0.93)***	0.96 (0.80–1.13)	0.81 (0.67–0.95)***
Mexico	1.91 (0.82–3.56)	1.95 (1.10–3.09)**	1.63 (1.03–2.19)**	1.24 (0.75–1.79)	2.29 (0.98–4.09)*	2.32 (1.16–3.83)**	1.66 (1.01–2.25)**	1.26 (0.67–1.93)
Russia	1.19 (0.61–1.93)	0.86 (0.41–1.53)	1.38 (0.83–1.89)	1.11 (0.68–1.58)	1.01 (0.54–1.67)	0.84 (0.42–1.45)	1.30 (0.78–1.82)	1.10 (0.67–1.56)
South Africa	0.41 (0.15–1.05)*	1.30 (0.77–2.00)	0.39 (0.15–0.89)**	1.37 (0.88–1.92)	0.51 (0.17–1.27)	1.38 (0.83–2.09)	0.45 (0.17–1.05)*	1.46 (0.92–2.05)
Pooled	0.83 (0.64–1.07)	0.82 (0.70–0.93)***	0.91 (0.75–1.09)	0.83 (0.73–0.94)***	0.96 (0.74–1.20)	0.95 (0.82–1.09)	1.02 (0.85–1.20)	0.94 (0.82–1.06)

*** p<0.01

** p<0.05

* p<0.1

#### Diet

The proportion of participants consuming 5 portions of fruit and vegetables per day was similar in migrant and rural groups in the pooled analyses. The urban group were more likely to consume daily recommended levels of fruit and vegetables, although this finding was only significant in the age and sex adjusted models (1.21 (1.06–1.36)). In the country level findings, fruit and vegetable consumption was higher in urban than in rural groups in India and Russia (age and sex adjusted RRs (1.75 (1.23–2.42); 2.77 (1.60–4.26); respectively). In contrast, the proportion of participants consuming 5 or more portions of fruit or vegetables was significantly higher in the migrant group (2.25 (1.04–3.24)) but not urban group, compared with the rural group in South Africa.

Regular alcohol consumption was significantly lower in the urban and migrant groups compared with the rural group in the pooled analyses (fully adjusted models (0.47 (0.31–0.68) and 0.58 (0.46–0.72) respectively). This pattern was seen in China and Ghana but not in the other countries. However in South Africa, urban participants were significantly more likely to consume alcohol regularly than the rural participants in the fully adjusted model (RR: 2.47 (1.05–5.09)) [S2 Fig; Table 4].

**Table 4:** Risk ratios for dietary variables in migrant and urban population groups with rural population groups as the reference category.

	Model 1: Adjusted for age and sex				Model 2: Adjusted for age, sex, income quintile, marital status and education			
	Fruit and veg consumption (> 5 portions per day)		Alcohol use (weekly or more frequently)		Fruit and veg consumption (> 5 portions per day)		Alcohol use (weekly or more frequently)	
	Migrant	Urban	Migrant	Urban	Migrant	Urban	Migrant	Urban
China	0.99 (0.89–1.04)	0.99 (0.92–1.03)	0.37 (0.20–0.67)***	0.38 (0.28–0.53)***	0.97 (0.85–1.03)	0.97 (0.89–1.02)	0.44 (0.24–0.79)***	0.46 (0.30–0.65)***
Ghana	0.92 (0.59–1.35)	1.17 (0.91–1.47)	0.59 (0.34–0.97)**	0.53 (0.37–0.74)***	0.90 (0.56–1.35)	1.13 (0.85–1.45)	0.70 (0.42–1.11)	0.63 (0.42–0.93)**
India	0.85 (0.50–1.40)	1.75 (1.23–2.42)***	1.21 (0.31–4.09)	0.60 (0.35–1.04)*	0.71 (0.44–1.13)	1.28 (0.88–1.83)	1.28 (0.32–4.46)	0.75 (0.42–1.31)
Mexico	0.68 (0.28–1.45)	1.29 (0.68–2.06)	0.81 (0.20–3.11)	0.94 (0.29–3.04)	0.76 (0.29–1.64)	1.46 (0.70–2.38)	0.77 (0.17–3.24)	1.08 (0.33–3.32)
Russia	1.83 (0.69–3.90)	2.77 (1.60–4.26)***	0.56 (0.14–2.04)	1.08 (0.49–2.22)	1.97 (0.71–4.23)	2.73 (1.56–4.24)***	0.43 (0.10–1.70)	1.06 (0.47–2.21)
South Africa	2.25 (1.04–3.24)**	1.34 (0.85–1.94)	0.28 (0.08–0.93)**	1.70 (0.66–3.92)	1.90 (0.81–3.01)	1.22 (0.69–1.92)	0.39 (0.10–1.38)	2.47 (1.05–5.09)**
Pooled	1.05 (0.79–1.31)	1.21 (1.06–1.36)***	0.41 (0.28–0.60)***	0.52 (0.42–0.64)***	0.97 (0.73–1.23)	1.14 (0.97–1.31)	0.47 (0.31–0.68)***	0.58 (0.46–0.72)***

***p<0.01

** p<0.05

* p<0.1

#### Physical Activity

Occupational physical activity was significantly lower in migrant and urban populations in the pooled analysis across the six countries (0.86 (0.72–0.98); 0.76 (0.65–0.85) respectively in the fully adjusted analysis). This pattern was evident across countries for urban groups, with statistically significant findings in China, Ghana, India and Russia. However, migrant populations in India and South Africa were most likely to be physically active at work (1.10 (1.01–1.17); 2.01 (1.53–2.28)). Conversely, leisure time physical activity was higher in migrant and urban groups in the pooled analyses (1.71 (1.11–2.53) and 2.36 (1.95–2.83) respectively in the fully adjusted analysis) with significant differences also seen in China, India and Russia.

Results for active travel were mixed. Although the pooled analyses suggest that there is increased active travel in urban and migrant populations (1.07 (1.01–1.12); 1.05 (1.00–1.09) respectively in the fully adjusted analysis), which was also demonstrated in individual country analyses in China, Russia and South Africa, urban populations were significantly less likely to travel using active transport in Ghana and India. Migrant groups showed a similar pattern to urban populations in the countries studied [S3 Fig; Table 5].

**Table 5:** Risk ratios for physical activity variables in migrant and urban population groups with rural population groups as the reference category.

	Model 1: Adjusted for age and sex					
	Occupational Physical Activity (>150 minutes)		Leisure time Physical Activity (>150 minutes)		Active Travel (>150 minutes)	
	Migrant	Urban	Migrant	Urban	Migrant	Urban
China	0.49 (0.26–0.76)	0.50 (0.35–0.68)	5.37 (3.09–8.37)	4.92 (3.36–6.89)	1.22 (1.12–1.29) ***	1.17 (1.07–1.26) ***
Ghana	0.73 (0.58–0.87)	0.67 (0.56–0.79)	1.28 (0.73–2.11)	1.20 (0.81–1.73)	0.94 (0.79–1.04)	0.87 (0.77–0.95)***
India	1.09 (0.99–1.16)	0.88 (0.81–0.95)	1.28 (0.76–2.00)	1.78 (1.43–2.18)	0.99 (0.93–1.05)	0.91 (0.83–0.97)***
Mexico	0.80 (0.38–1.32)	0.82 (0.52–1.18)	2.49 (0.65–7.57)	2.19 (0.71–5.84)	1.00 (0.75–1.15)	0.94 (0.73–1.09)
Russia	0.96 (0.79–1.06)	0.87 (0.71–1.00)	0.54 (0.18–1.44)	2.88 (1.32–5.22)	1.05 (0.88–1.14)	1.08 (0.98–1.14)
South Africa	2.08 (1.67–2.30)	1.07 (0.71–1.46)	0.96 (0.26–2.94)	1.39 (0.64–2.77)	1.15 (0.88–1.26)	1.16 (1.05–1.23)***
Pooled	0.80 (0.65–0.94)***	0.67 (0.57–0.78)***	1.88 (1.22–2.75)***	2.68 (2.23–3.17)***	1.06 (1.00–1.11)*	1.03 (0.99–1.08)
	Model 2: Adjusted for age, sex, income quintile, marital status and education					
	Occupational Physical Activity (>150 minutes)		Leisure time Physical Activity (>150 minutes)		Active Travel (>150 minutes)	
China	0.59 (0.35–0.87)***	0.62 (0.44–0.81)***	5.10 (3.02–7.86)***	4.52 (3.07–6.37)***	1.22 (1.12–1.30) ***	1.18 (1.07–1.26) ***
Ghana	0.78 (0.62–0.90)***	0.72 (0.60–0.83)***	0.91 (0.48–1.60)	0.88 (0.53–1.39)	0.95 (0.80–1.04)	0.88 (0.76–0.97)***
India	1.10 (1.01–1.17)**	0.94 (0.87–1.00)*	1.24 (0.74–1.95)	1.60 (1.28–1.98)***	1.00 (0.93–1.05)	0.91 (0.83–0.98)***
Mexico	0.76 (0.35–1.31)	0.75 (0.45–1.14)	2.71 (0.60–8.92)	1.90 (0.55–5.64)	1.02 (0.75–1.16)	0.98 (0.76–1.12)
Russia	0.97 (0.81–1.07)	0.86 (0.69–0.98)**	0.74 (0.24–2.08)	3.13 (1.40–5.63)***	1.05 (0.88–1.14)	1.09 (0.99–1.15)*
South Africa	2.01 (1.53–2.28)***	1.02 (0.63–1.47)	1.04 (0.30–3.08)	1.21 (0.51–2.62)	1.15 (0.86–1.27)	1.17 (1.05–1.24)**
Pooled	0.86 (0.72–0.98)**	0.76 (0.65–0.85)***	1.71 (1.11–2.53)**	2.36 (1.95–2.83)***	1.07 (1.01–1.12)**	1.05 (1.00–1.09)*

*** p<0.01

** p<0.05

* p<0.1

#### Obesity

Overweight measured by BMI and raised waist circumference were significantly higher in urban populations compared with rural populations in the pooled analyses (RR 1.19 (1.04–1.35) and RR 1.24 (1.07–1.42) respectively). Indicators of obesity were generally worse in urban and migrant populations and many of these reached significance for urban and migrant populations in Ghana and India. However Russia was an exception, with all indicators showing lower levels of obesity in urban and migrant populations compared with rural populations, and significantly lower obesity measured using BMI for both migrant and urban populations (0.47 (0.23–0.87); 0.42(0.22–0.75)) [S4 Fig; Table 6].

**Table 6:** Risk ratios for obesity variables in migrant and urban population groups with rural population groups as the reference category.

	Model 1: Adjusted for age and sex					
	Obesity (BMI ≥30)		Overweight (BMI ≥25)		Raised waist circumference (>94cm for men; >80cm for women)	
	Migrant	Urban	Migrant	Urban	Migrant	Urban
China	1.18 (0.55–2.43)	1.10 (0.67–1.79)	1.00 (0.71–1.33)	1.03 (0.82–1.27)	1.03 (0.66–1.46)	1.27 (0.94–1.62)
Ghana	2.51 (1.13–5.03)**	3.17 (2.03–4.75)***	2.08 (1.47–2.75)***	2.35 (1.91–2.79)***	1.60 (1.13–2.08)**	1.63 (1.33–1.92)***
India	0.80 (0.45–1.39)	1.24 (0.80–1.89)	1.54 (1.19–1.98)***	1.88 (1.47–2.40)***	1.55 (1.23–1.91)***	1.58 (1.32–1.86)***
Mexico	1.68 (0.96–2.55)*	1.30 (0.72–2.08)	1.01 (0.80–1.12)	0.90 (0.73–1.03)	1.00 (0.67–1.21)	0.91 (0.66–1.12)
Russia	0.48 (0.24–0.88)**	0.45 (0.24–0.78)***	0.65 (0.37–0.96)**	0.86 (0.60–1.10)	0.75 (0.42–1.12)	0.86 (0.57–1.14)
South Africa	0.86 (0.30–1.79)	0.99 (0.67–1.38)	1.41 (1.00–1.62)*	1.08 (0.81–1.32)	1.23 (0.70–1.62)	1.07 (0.74–1.37)
Pooled	1.16 (0.79–1.68)	1.14 (0.88–1.47)	1.18 (0.95–1.45)	1.19 (1.04–1.35)**	1.20 (0.99–1.44)*	1.24 (1.07–1.42)***
	Model 2: Adjusted for age, sex, income quintile, marital status and education					
	Obesity (BMI ≥30)		Overweight (BMI ≥25)		Raised waist circumference (>94cm for men; >80cm for women)	
China	1.24 (0.58–2.54)	1.16 (0.68–1.95)	0.99 (0.71–1.32)	1.05 (0.83–1.28)	0.94 (0.64–1.30)	1.17 (0.92–1.46)
Ghana	1.86 (0.82–3.90)	2.38 (1.44–3.78)***	1.71 (1.15–2.37)**	1.97 (1.52–2.44)***	1.39 (0.96–1.85)*	1.40 (1.11–1.71)***
India	0.73 (0.41–1.29)	1.14 (0.72–1.77)	1.35 (1.05–1.71)**	1.50 (1.15–1.94)***	1.44 (1.19–1.72)***	1.35 (1.12–1.60)***
Mexico	1.56 (0.88–2.42)	1.36 (0.71–2.25)	1.01 (0.80–1.13)	0.95 (0.79–1.06)	1.03 (0.68–1.24)	1.01 (0.79–1.17)
Russia	0.47 (0.23–0.87)**	0.42 (0.22–0.75) ***	0.67 (0.40–0.98)**	0.84 (0.57–1.10)	0.76 (0.41–1.13)	0.86 (0.56–1.16)
South Africa	0.95 (0.39–1.76)	0.85 (0.55–1.23)	1.38 (0.93–1.62)*	1.03 (0.72–1.30)	1.30 (0.78–1.66)	1.06 (0.69–1.40)
Pooled	1.16 (0.79–1.68)	1.14 (0.88–1.47)	1.18 (0.95–1.45)	1.19 (1.04–1.35)*	1.20 (0.99–1.44)	1.24 (1.07–1.42)**

*** p<0.01

** p<0.05

* p<0.1

#### Hypertension and diabetes

There was no obvious association between prevalence of hypertension and urban, rural or migrant status, and pooled analyses showed no significant association when country data were combined. Prevalence of hypertension was significantly lower in migrant and urban groups in China compared with rural groups (0.72 (0.55–0.91); 0.83 (0.68–0.99)), however the reverse was true in Ghana and India where hypertension prevalence was significantly higher in urban groups compared with rural groups (1.19 (0.97–1.41); 1.28(1.03–1.55)). Migrant groups in Mexico had significantly higher prevalence of hypertension than rural groups (1.46 (0.97–1.97)), whereas in South Africa this was reversed (0.71 (0.34–1.12)). [S5 Fig; Table 7].

**Table 7:** Risk ratios for disease variables in migrant and urban population groups with rural population groups as the reference category.

	Model 1: Adjusted for age and sex				Model 2: Adjusted for age, sex, income quintile, marital status and education			
	Hypertension (Systolic ≥140, diastolic ≥90 or on antihypertensive medication)		Diabetes (self-report of doctor-diagnosis)		Hypertension (Systolic ≥140, diastolic ≥90 or on antihypertensive medication)		Diabetes (self-report of doctor-diagnosis)	
	Migrant	Urban	Migrant	Urban	Migrant	Urban	Migrant	Urban
China	0.73 (0.54–0.95)**	0.86 (0.69–1.04)	1.86 (1.06–3.24)**	2.20 (1.33–3.58)***	0.72 (0.55–0.91)***	0.83 (0.68–0.99)**	1.86 (1.05–3.25)**	2.18 (1.35–3.47)***
Ghana	1.18 (0.87–1.51)	1.25 (1.05–1.45)**	9.50 (3.27–25.6)***	3.57 (1.66–7.57)***	1.12 (0.80–1.45)	1.19 (0.97–1.41)*	5.15 (1.89–13.5)***	1.89 (0.82–4.27)
India	0.93 (0.73–1.18)	1.30 (1.06–1.56)**	1.14 (0.60–2.15)	1.86 (1.24–2.75)***	0.91 (0.71–1.15)	1.28 (1.03–1.55)**	0.96 (0.53–1.74)	1.44 (0.95–2.18)*
Mexico	1.44 (0.99–1.91)*	0.99 (0.66–1.38)	1.33 (0.42–3.67)	1.39 (0.49–3.51)	1.46 (0.97–1.97)*	1.02 (0.65–1.48)	1.07 (0.29–3.44)	1.31 (0.37–3.97)
Russia	0.85 (0.51–1.23)	0.87 (0.52–1.26)	1.25 (0.66–2.32)	1.06 (0.55–1.99)	0.83 (0.50–1.22)	0.86 (0.51–1.26)	1.24 (0.65–2.29)	1.01 (0.52–1.91)
South Africa	0.57 (0.24–1.03)*	0.93 (0.68–1.17)	1.42 (0.55–3.54)	3.36 (1.92–5.74)***	0.71 (0.34–1.12)*	0.94 (0.64–1.21)	1.60 (0.66–3.77)	3.48 (1.64–7.09)***
Pooled	0.91 (0.76–1.09)	0.97 (0.86–1.09)	1.79 (1.19–2.65)***	1.88 (1.36–2.60)***	0.95 (0.79–1.12)	0.99 (0.87–1.12)	1.60 (1.04–2.43)**	1.69 (1.15–2.47)***

*** p<0.01

** p<0.05

* p<0.1

In contrast, diagnosed diabetes showed a very consistent association across all the countries studied with significantly higher prevalence of diagnosed diabetes in migrant and urban groups in the pooled analyses (1.60 (1.04–2.43); 1.69 (1.15–2.47). This association was also significant for urban groups in the country-specific analyses for China, India and South Africa (2.18 (1.35–3.47); 1.44 (0.95–2.18); 3.48 (1.64–7.09)) and for migrant groups in China and Ghana (1.86 (1.05–3.25); 5.15 (1.89–13.50)) [S5 Fig; Table 7].

## Discussion

### Principal findings

Migrants and urban dwellers had similar NCD risk profiles although these were not consistently worse than that seen in rural dwellers. Regular alcohol consumption was lower in urban and migrant groups than in rural groups. Occupational physical activity was lower in urban and migrant groups while active travel and recreational physical activity were higher. Overweight, raised waist circumference and diagnosed diabetes were higher in urban groups. There were some important country level exceptions to these trends. Obesity indicators were higher in rural groups in Russia; active travel was lower in urban groups in Ghana and India; occupational physical activity was highest in the migrant group in India and South Africa; and in South Africa, urban groups had the highest alcohol consumption. Urban, migrant and rural patterns of smoking and hypertension prevalence varied between countries.

### Previous studies

Our findings on obesity are consistent with those from a recent systematic review which found that obesity was highest in urban and lowest in rural groups, with migrants at an intermediate level.[16] This is likely to be due to a different energy balance in urban dwellers compared with rural dwellers, potentially due to the lower levels of occupational physical activity by urban dwellers identified in our study, perhaps as well as increased calorie intake. The review also found that there was no consistent association between migration and hypertension status. This may be because the studies identified by the systematic review were heterogeneous in design, including country setting. We also found differing results in the country-specific analyses conducted in this study, and no significant result when country data were pooled. Hypertension is a complex condition with many contributing risk factors including environmental (eg: air pollution; stressful living conditions), lifestyle (eg: smoking and obesity rates) and population genetics. Country level differential exposure to these risk factors in urban and rural dwellers may explain the inconsistent association between migration status and hypertension.

Studies carried out in India have reported lower prevalence of diabetes in rural areas,[21, 22] and that physical activity [13, 22] and active travel [23] are higher in rural dwellers than migrant or urban groups. These findings are consistent with our India-specific results, although the results for active travel in other countries differ.

Studies of smoking and urbanisation have shown conflicting results which may reflect heterogeneity in sampling. For example, while some studies suggest that migrant and urban groups are less likely to smoke than rural groups in India and China [22, 24, 25] other studies report the opposite.[13, 26, 27] There are mixed results from studies of smoking behaviour in other countries.[28–31] The countries studied are at different stages of the tobacco epidemic and may vary in their implementation of effective tobacco control strategies in urban and rural settings [32]. It may be that in some countries, or in some areas, urban dwellers are more likely to be exposed to cigarette advertising, whereas in other areas urban dwellers are more likely to be exposed to health messages highlighting the dangers of smoking. Finally, there is very little evidence on patterning of alcohol or fruit and vegetable consumption by location and migrant status in LMICs.

### Strengths and limitations

This study uses nationally representative data from six populous middle-income countries experiencing rapid economic growth, urbanisation and increasing NCD risk. Survey data were collected using consistent tools and measures, including objective measures of anthropometry and blood pressure, allowing robust cross country comparisons. However, the survey was designed to focus on the health of older populations and for this reason there are many more observations of individuals from older age-groups and the sample of individuals aged 18–49 is smaller. In addition, a number of outcome measures were based on self-report and may be subject to biases, including social desirability bias, as well as error. In particular, we noted that Russia had a lower prevalence of frequent drinking than might be expected. A qualitative study has suggested that drinking can be under-reported in Russia where small amounts of alcohol, especially beer, may not be perceived as a drinking event.[33] It may be that there are other cultural differences that alter the way risk-factors are reported.

We were unable to look at dietary measures other than fruit and vegetable consumption and alcohol intake. More detailed information on consumption of other food groups and fat, sugar and salt intakes would provide a clearer picture of the impact of migration on nutrition. Small sample sizes meant that some of our country level estimates lacked precision, particularly in the migrant groups. Pooled estimates were used to address this, however these may mask between country heterogeneity. We used country specific definitions of urban and rural location and this variable was dichotomous. There is a growing literature suggesting that this distinction is overly simplistic, as aspects of urban living develop in rural areas.[13, 34] Finally, we were unable to examine time period of exposure to the urban environment among the migrant group, this means we are unable to assess what differences might exist between recent arrivals to the urban environment and longer-term residents of urban areas. We also did not take account of urban-urban migrants in our analyses (treating these participants as urban dwellers). These intra and inter-urban movements may also be associated with changing exposure to NCD risk-factors [35] however current projections demonstrate that migration from rural to urban areas requires attention as a large shift in population, which potentially has a greater effect on health behaviour, and this is the focus of this paper. A pragmatic approach using well-defined groups may be required to inform health policy.

### Policy implications

This study suggests that it is simplistic to assume that urban populations adopt a less healthy lifestyle than rural populations. In fact urban populations were more likely to be physically active in their leisure time and for travel, they were also generally less likely to drink alcohol regularly and in some countries were less likely to smoke. To address non-communicable disease in the whole population, different strategies may be needed in rural and urban areas recognising the differing risk-factor profiles of these groups. Tobacco use in rural areas is particularly interesting. Tobacco products are being heavily promoted in LMICs and the prevalence in rural areas may indicate market penetration is occurring throughout LMICs, as has been documented with processed foods.[36]

We have demonstrated that middle income countries are not all facing the same health challenges, for example although rural populations are less likely to be obese in Ghana and India they are more likely to be obese in Russia, and while regular alcohol drinking is generally lower in urban populations, in South Africa urban populations are more likely to drink regularly. These findings indicate that a ‘one size fits all’ approach to addressing the growing burden of NCDs in middle income countries may not be appropriate. It can be convenient for policy-makers to use other countries with a similar level of economic development as a model for their own, but timely health intelligence on the national or sub-national distribution of risk-factors is necessary to inform preventative strategies.

Diagnosed diabetes prevalence is higher in urban groups and this trend is seen across all six countries. It may be that this is due to under-diagnosis in rural areas perhaps due to a factor associated with rurality, such as distance to services, which would suggest increased access to service, perhaps through telemedicine, could reduce inequality. It may also be due to the increased obesity generally seen in urban groups.

Rural-urban migrants had broadly similar risk-factor profiles to the urban group suggesting that exposure to urban environments may promote assimilation of health behaviour regardless of previous life experiences. The combination of cross-sectional design of the SAGE study and that the dataset lacked detailed information about timing of migration means we are not able to determine when transition to urban risk profile occurs in migrants. However previous studies suggest this occurs in the first ten years after migration.[37] Therefore, interventions to promote retention of health behaviours associated with rural life need to be targeted soon after migration occurs.

## Conclusion

Migrants and urban dwellers had similar NCD risk-factor profiles which were not consistently worse than those seen in rural dwellers. The variable impact of urbanisation on risk-factor profiles, and marked between country heterogeneity, should be considered in the design and evaluation of strategies to achieve the WHO 25x25 target.

## Supporting Information

S1 Fig Prevalence of current and ever smoking in rural, migrant and urban groups. (TIF)

S2 Fig Prevalence of adequate fruit or vegetables consumption (more than 5 pieces per day) and of frequent alcohol consumption (weekly or more) in rural, migrant and urban groups. (TIF)

S3 Fig Prevalence of adequate physical activity (150 minutes or more per week) met through occupational or leisure time physical activity or through active travel in rural, migrant and urban groups. (TIF)

S4 Fig Prevalence of obesity (BMI ≥ 30), overweight (BMI ≥ 25) and raised waist circumference (≥ 80cm for women, ≥ 94cm for men) in rural, migrant and urban groups. (TIF)

S5 Fig Prevalence of hypertension (systolic blood pressure ≥ 140 mmHg, diastolic ≥ 90 mmHg or on blood pressure medication) and doctor-diagnosed diabetes in rural, migrant and urban groups. (TIF)

## References

[1] Lozano R, Naghavi M, Foreman K, Lim S, Shibuya K, Aboyans V, et al Global and regional mortality from 235 causes of death for 20 age groups in 1990 and 2010: a systematic analysis for the Global Burden of Disease Study 2010. Lancet. 2012 12;380(9859):2095–2128 10.1016/S0140-6736(12)61728-0 23245604 PMC10790329

[2] Murray CJ, Vos T, Lozano R, Naghavi M, Flaxman AD, Michaud C, et al Disability-adjusted life years (DALYs) for 291 diseases and injuries in 21 regions, 1990–2010: a systematic analysis for the Global Burden of Disease Study 2010. Lancet. 2012 12;380(9859):2197–2223. 10.1016/S0140-6736(12)61689-4 23245608

[3] WHO. Global status report on noncommunicable diseases 2010: Description of the global burden of NCDs, their risk-factors and determinants. 4 2011

[4] United Nations General Assembly. Resolution adopted by the General Assembly: Political Declaration of the High—level Meeting of the General Assembly on the Prevention and Control of Non-communicable Diseases. 3^rd^ plenary meeting. 19^th^ September 2011. Available: http://www.who.int/nmh/events/un_ncd_summit2011/political_declaration_en.pdf.

[5] World Health Organisation. NCD Global Monitoring Framework. 2013. Available: http://www.who.int/nmh/global_monitoring_framework/en/.

[6] UN Department of Economic and Social Affairs, Population Division. World Urbanization Prospects, the 2011 Revision. Available: http://esa.un.org/unup/.

[7] Yusuf F, Saich T. China urbanizes: consequences, strategies and policies. The World Bank, Washington, DC (2008).

[8] Harpham T. (2009). Urban health in developing countries: What do we know and where do we go? Health and Place, 15(1), 107–116. 10.1016/j.healthplace.2008.03.004 18455952

[9] Lu Y. Test of the 'healthy migrant hypothesis': a longitudinal analysis of health selectivity of internal migration in Indonesia. Social science & medicine (1982). 2008 10;67(8):1331–1339. 18639967 10.1016/j.socscimed.2008.06.017

[10] Misch KA. Ischaemic heart disease in urbanized Papua New Guinea. An autopsy study. Cardiology. 1988;75(1):71–75. 3342427 10.1159/000174351

[11] Wagner KHH, Brath H. A global view on the development of non communicable diseases. Preventive medicine. 2012 5;54 Suppl. 10.1016/j.ypmed.2011.11.012 22178469

[12] Patel RB, Burke TF. Urbanization-an emerging humanitarian disaster. The New England journal of medicine. 2009 8;361(8):741–743. 10.1056/NEJMp0810878 19692687

[13] Allender S, Lacey B, Webster P, Rayner M, Deepa M, Scarborough P, et al Level of urbanization and noncommunicable disease risk-factors in Tamil Nadu, India. Bulletin of the World Health Organization. 2010 4;88(4):297–304. 10.2471/BLT.09.065847 20431794 PMC2855597

[14] Kyobutungi C, Egondi T, Ezeh A. The health and well-being of older people in Nairobi's slums. Global health action. 2010;3 10.3402/gha.v3i0.2138 PMC2957141 20959873

[15] Allender S, Lacey B, Webster P, Rayner M, Deepa M, Scarborough P, et al Level of urbanization and noncommunicable disease risk-factors in Tamil Nadu, India. Bulletin of the World Health Organization. 2010 4;88(4):297–304 10.2471/BLT.09.065847 20431794 PMC2855597

[16] Hernández AV, Pasupuleti V, Deshpande A, Bernabé-Ortiz A, Miranda JJ. Effect of rural-to-urban within-country migration on cardiovascular risk-factors in low- and middle-income countries: a systematic review. Heart. 2012 2;98(3):185–194. 10.1136/heartjnl-2011-300599 21917659 PMC3272377

[17] Zaman MJ. Migrating towards a heart attack. Heart (British Cardiac Society). 2012 2;98(4):271–273. 10.1136/heartjnl-2011-301200 22107758

[18] Kontis V, Mathers CD, Rehm J, Stevens GA, Shield KD, Bonita R, et al Contribution of six risk-factors to achieving the 25×25 non-communicable disease mortality reduction target: a modelling study. Lancet. 2014 5 10.1016/S0140-6736(14)60616-4 24797573

[19] Kowal P, Chatterji S, Naidoo N, Biritwum R, Fan W, Lopez Ridaura R, et al Data resource profile: the World Health Organization Study on global AGEing and adult health (SAGE). International journal of epidemiology. 2012 12;41(6):1639–1649. 10.1093/ije/dys210 23283715 PMC3535754

[20] Zhang J, Yu KF. What's the relative risk? A method of correcting the odds ratio in cohort studies of common outcomes. JAMA: the journal of the American Medical Association. 1998 11;280(19):1690–1691. 9832001 10.1001/jama.280.19.1690

[21] Mohan V, Mathur P, Deepa R, Deepa M, Shukla DK, Menon GR, et al Urban rural differences in prevalence of self-reported diabetes in India-the WHO-ICMR Indian NCD risk-factor surveillance. Diabetes research and clinical practice. 2008 4;80(1):159–168. 10.1016/j.diabres.2007.11.018 18237817

[22] Ebrahim S, Kinra S, Bowen L, Andersen E, Ben-Shlomo Y, Lyngdoh T, et al The effect of rural-to-urban migration on obesity and diabetes in India: a cross-sectional study. PLoS medicine. 2010 4;7(4):e1000268+ 10.1371/journal.pmed.1000268 20436961 PMC2860494

[23] Millett C, Agrawal S, Sullivan R, Vaz M, Kurpad A, Bharathi AV, et al Associations between Active Travel to Work and Overweight, Hypertension, and Diabetes in India: A Cross-Sectional Study. PLoS Med. 2013 6;10(6):e1001459+ 10.1371/journal.pmed.1001459 23776412 PMC3679004

[24] Chen X, Li X, Stanton B, Fang X, Lin D, Cole M, et al Cigarette smoking among rural-to-urban migrants in Beijing, China. Preventive medicine. 2004 10;39(4):666–673. 15351531 10.1016/j.ypmed.2004.02.033

[25] Mou J, Fellmeth G, Griffiths S, Dawes M, Cheng J. Tobacco smoking among migrant factory workers in Shenzhen, China. Nicotine & tobacco research. 2013 1;15(1):69–76. 22492086 10.1093/ntr/nts085

[26] Yang T, Wu J, Rockett IR, Abdullah AS, Beard J, Ye J. Smoking patterns among Chinese rural-urban migrant workers. Public health. 2009 11;123(11):743–749. 10.1016/j.puhe.2009.09.021 19896682

[27] Chen X, Li X, Stanton B, Fang X, Lin D, Cole M, et al Cigarette smoking among rural-to-urban migrants in Beijing, China. Preventive medicine. 2004 10;39(4):666–673. 15351531 10.1016/j.ypmed.2004.02.033

[28] Vorster HH. The emergence of cardiovascular disease during urbanisation of Africans. Public health nutrition. 2002 2;5(1A):239–243. 12027290 10.1079/phn2001299

[29] Williams CT, Grier SA, Marks ASS. "Coming to town": the impact of urbanicity, cigarette advertising, and network norms on the smoking attitudes of black women in Cape Town, South Africa. Journal of urban health: bulletin of the New York Academy of Medicine. 2008 7;85(4):472–485. 10.1007/s11524-008-9286-7 18563573 PMC2443257

[30] Peer N, Bradshaw D, Laubscher R, Steyn N, Steyn K. Urban-rural and gender differences in tobacco and alcohol use, diet and physical activity among young black South Africans between 1998 and 2003. Global health action. 2013;6. 10.3402/gha.v6i0.19216 PMC3559753 23364100

[31] Nguyen LT, Rahman Z, Emerson MR, Nguyen MH, Zabin LSS. Cigarette smoking and drinking behavior of migrant adolescents and young adults in Hanoi, Vietnam. The Journal of adolescent health. 2012 3;50(3 Suppl) 10.1016/j.jadohealth.2011.12.010 22340858 PMC4169841

[32] Thun M, Peto R, Boreham J, Lopez AD. Stages of the cigarette epidemic on entering its second century. Tobacco Control. 2012 3;21(2):96–101. 10.1136/tobaccocontrol-2011-050294 22345230

[33] Bobrova N, West R, Malyutina D, Malyutina S, Bobak M. Gender differences in drinking practices in middle aged and older Russians. Alcohol and alcoholism (Oxford, Oxfordshire). 2010;45(6):573–580. 10.1093/alcalc/agq069 21075855 PMC3943390

[34] Dahly DL, Adair LS. Quantifying the urban environment: a scale measure of urbanicity outperforms the urban-rural dichotomy. Social science & medicine (1982). 2007 4;64(7):1407–1419. 17196724 10.1016/j.socscimed.2006.11.019 PMC2001275

[35] Vearey J, Palmary I, Thomas L, Nunez L, Drimie S. Urban health in Johannesburg: the importance of place in understanding intra-urban inequalities in a context of migration and HIV. Health & place. 2010 7;16(4):694–702. 20400354 10.1016/j.healthplace.2010.02.007

[36] Stuckler D, Nestle M. Big food, food systems, and global health. PLoS medicine. 2012;9(6). 10.1371/annotation/4c5252d3-2fd5-4ff2-b3d9-ed197690f877 22723746 PMC3378592

[37] Kinra S, Andersen E, Ben-Shlomo Y, Bowen L, Lyngdoh T, Prabhakaran D, et al Association between urban life-years and cardiometabolic risk: the Indian migration study. American journal of epidemiology. 2011 7;174(2):154–164. 10.1093/aje/kwr053 21622949 PMC3132275

