# Supplementary figures and images for: Rural, Urban and Migrant Differences in Non-Communicable Disease Risk-Factors in Middle Income Countries: A Cross-Sectional Study of WHO-SAGE Data
Source: PLoS One. 2015 Apr 7;10(4):e0122747. doi: 10.1371/journal.pone.0122747 (PMC4388413; doi:10.1371/journal.pone.0122747)

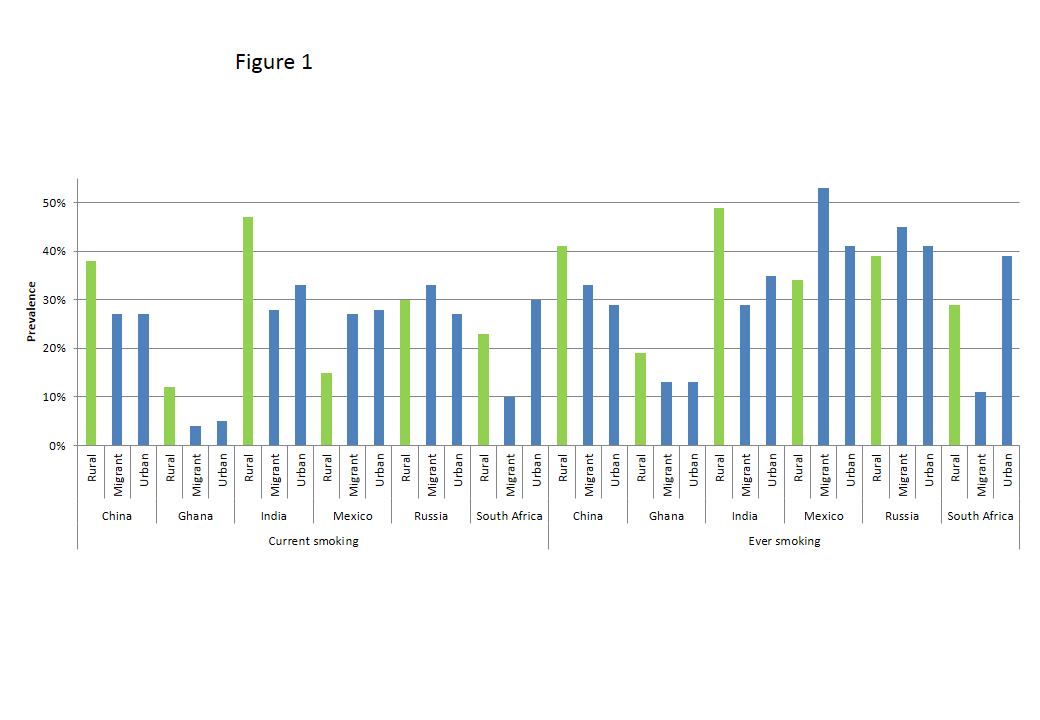

Supplement: S1 Fig — (TIF) [file pone.0122747.s001.TIF]

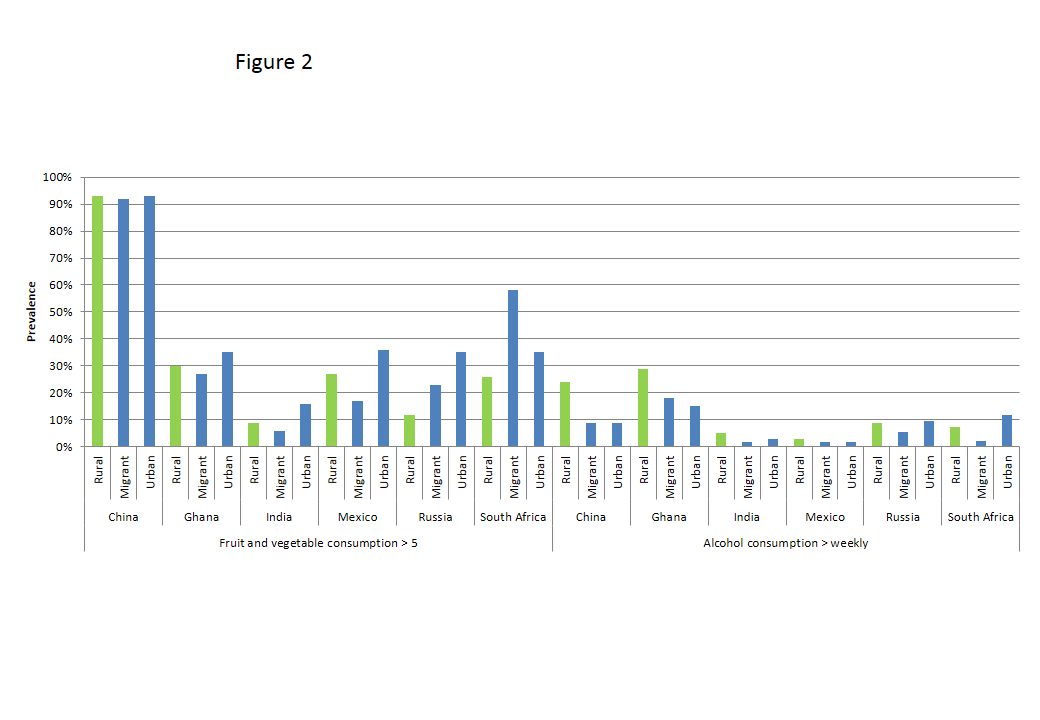

Supplement: S2 Fig — (TIF) [file pone.0122747.s002.TIF]

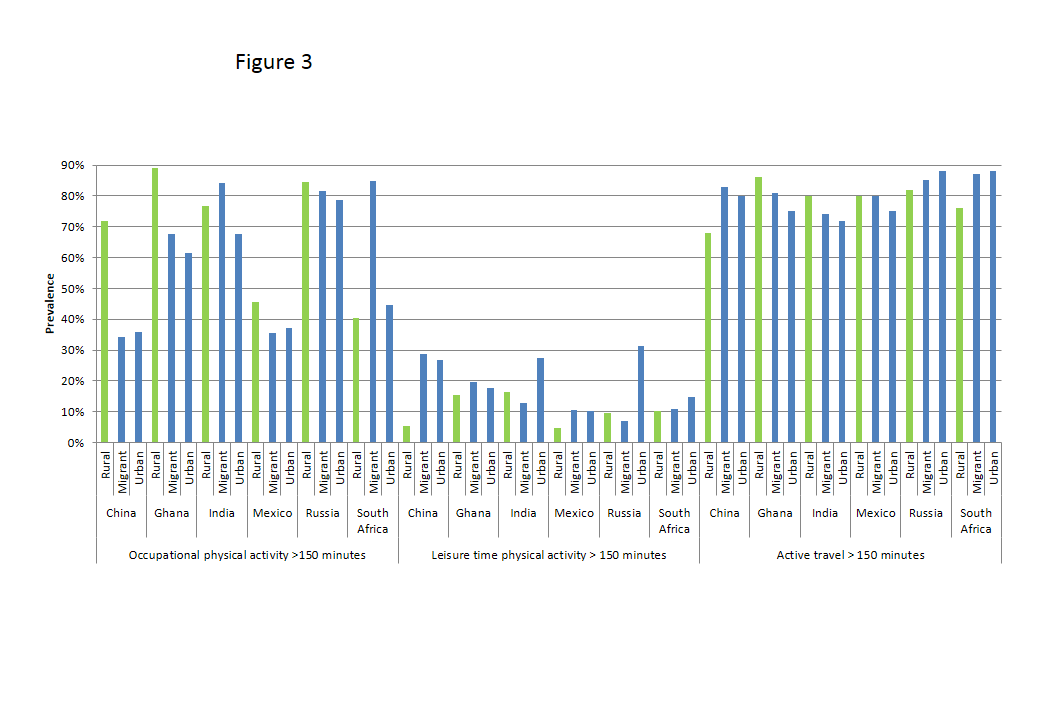

Supplement: S3 Fig — (TIF) [file pone.0122747.s003.TIF]

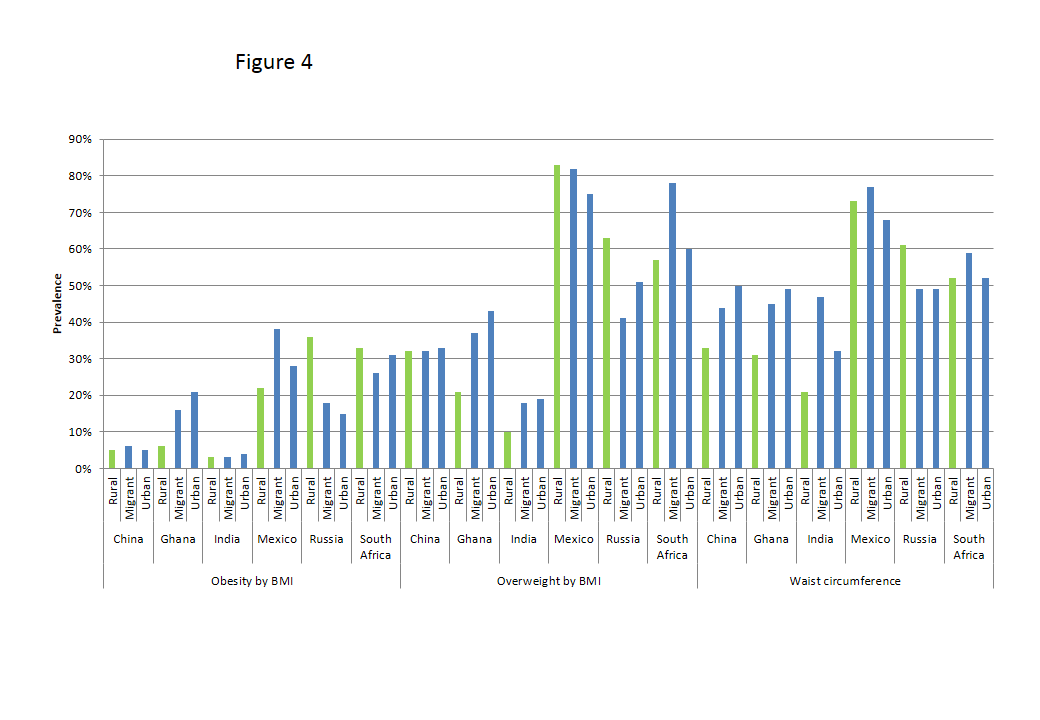

Supplement: S4 Fig — (TIF) [file pone.0122747.s004.TIF]

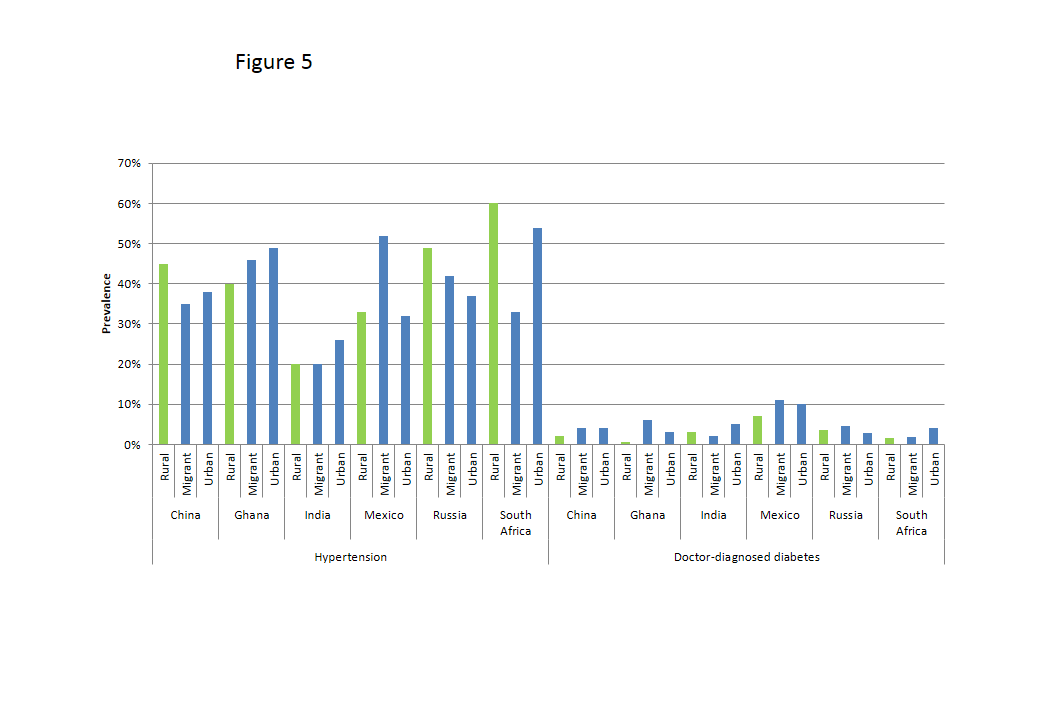

Supplement: S5 Fig — (TIF) [file pone.0122747.s005.TIF]
